# An ideal-typical model for comparing interprofessional relations and skill mix in health care

**DOI:** 10.1186/s12913-016-1881-9

**Published:** 2016-11-08

**Authors:** Walter Schönfelder, Elin Anita Nilsen

**Affiliations:** 1 Institute for Child Welfare Services and Social Work, UiT The Arctic University of Norway, Breivika, Tromsø, 9037 Norway; 2 School of Business and Economics, UiT The Arctic University of Norway, Breivika, Tromsø, 9037 Norway

**Keywords:** Comparative health services research, Interprofessional relations, Professional jurisdiction, Nursing education, Professional autonomy, Professional hierarchy, Skill mix, Interdisciplinary teamwork, Health care, Social care, Ideal type

## Abstract

**Background:**

Comparisons of health system performance, including the regulations of interprofessional relations and the skill mix between health professions are challenging. National strategies for regulating interprofessional relations vary widely across European health care systems. Unambiguously defined and generally accepted performance indicators have to remain generic, with limited power for recognizing the organizational structures regulating interprofessional relations in different health systems. A coherent framework for in-depth comparisons of different models for organizing interprofessional relations and the skill mix between professional groups is currently not available. This study aims to develop an ideal-typical framework for categorizing skill mix and interprofessional relations in health care, and to assess the potential impact for different ideal types on care coordination and integrated service delivery.

**Methods:**

A document analysis of the Health Systems in Transition (HiT) reports published by the European Observatory on Health Systems and Policies was conducted. The HiT reports to 31 European health systems were analyzed using a qualitative content analysis and a process of meaning condensation.

**Results:**

The educational tracks available to nurses have an impact on the professional autonomy for nurses, the hierarchy between professional groups, the emphasis given to negotiating skill mix, interdisciplinary teamwork and the extent of cooperation across the health and social service interface. Based on the results of the document analysis, three ideal types for regulating interprofessional relations and skill mix in health care are delimited. For each ideal type, outcomes on service coordination and holistic service delivery are described.

**Conclusions:**

Comparisons of interprofessional relations are necessary for proactive health human resource policies. The proposed ideal-typical framework provides the means for in-depth comparisons of interprofessional relations in the health care workforce beyond of what is possible with directly comparable, but generic performance indicators.

## Background

The need for international comparisons of health systems performance is a much-discussed topic in health policy planning, just as the problematic nature of such comparisons has been pointed out repeatedly. In a policy summary issued on behalf of the World Health Organization (WHO) an argument is made for founding international comparisons on performance indicators with “widespread acceptance … defined in unambiguous terms” ([1]: IV). As appealing as an ideal, the ability of unambiguously defined indicators to capture and directly compare key elements of health system performance is limited. In order to be precisely measurable, such indicators have to remain generic, with limited potential for recognizing the different organizational structures regulating health service delivery. This is particularly true for comparisons of health human resource (HHR) policy, since national policies governing interprofessional relations and the skill mix among health-professions vary significantly between countries.

In HHR planning, the readjustment of the extent of professional autonomy for different health professions has become an often and sometimes quite heatedly debated topic for HHR policy and the provision of effective services [2–4]. The debate is all but uncontroversial, since the term skill mix is used with a variety of meanings. Sibbald et.al. proposed a helpful general framework for distinguishing concepts of skill mix focusing on changing professional roles and those focusing on changing relations between services [5]. Recently, Bourgeault and Merritt proposed another conceptual framework embracing micro, meso and macro factors affecting the scopes of practice for health professionals [6]. Both frameworks give valuable input for assessing the conditions and consequences of adjusting the skill mix between professions. The question is how sensitive these frameworks are to embrace specific context-dependent factors regulating the skill mix in different health systems.

### Aims and objectives

We want with our study to address this gap in research based knowledge. We first illustrate the problematic nature of precisely measurable and directly comparable performance indicators from the perspective of HHR-policy before introducing the concept of professional jurisdiction as the theoretical background for comparing skill mix and interprofessional relations in 31 European health care systems. Based on this comparison, our aims are to develop an ideal-typical framework for categorizing skill mix and interprofessional relations in health care, and to assess the potential impact for different ideal types on care coordination and integrated service delivery.

### Challenges for comparing interprofessional relations in health care

A frequently used indicator in comparative health service research is the ratio between practicing physicians and nurses. This ratio is precisely measurable, widely accepted and quite unambiguously defined. It is commonly used for comparing human resource allocation in health care and as a performance indicator for health service delivery [7, 8]. However, for the purpose of in-depth cross-national comparisons, this ratio reveals little about the actual performance of nurses and physicians in a given health system, for example in terms of accessibility to and quality of care. To that purpose, additional indicators, such as the allocation of nurses and physicians between primary and specialist health services will have to be employed, in which case the demand for an unambiguous definition is rapidly losing shape since the organizational boundaries between primary and specialized care vary widely between countries.

Even more challenging to apply are qualitative indicators with a potential to provide comparable information about the practical tasks performed by different occupational groups, or the relative autonomy for each group in the execution of their tasks. The importance of these indicators becomes evident with an example. The ratio of nurses and physicians is almost identical in Sweden (2.9) and Germany (3.0) [9]. Nevertheless, for comparative purposes it would be inadequate to infer from the similar ratio a similar quality in health system performance since both the level of service delivery for nurses as well as the professional autonomy in the performance of their tasks are regulated quite differently in the two countries. In Sweden, a large number of nurses is employed in the primary health sector, with community (or district) nurses as a basic pillar of the primary health service and with a high degree of professional autonomy for the nursing workforce [10]. In Germany, the large majority of nurses is employed in the specialized health services, with community nursing still a field in its very infancy. In both sectors of the German health care system, the professional authority of nurses is subordinated to those of the medical profession [11, 12]. This example illustrates on the one hand the importance of interprofessional relations as a core ingredient for HHR policy. On the other hand, it emphasizes the importance of qualitative performance indicators in cross-national comparisons of interprofessional relations in health care. Comparisons of the nursing staff in the health care workforce are a particularly challenging task because, as pointed out by McKee et.al., the term nurse in itself is rather a misnomer that obscures “the tremendous diversity in roles that are associated with them” ([13]: 65).

The need for more coordination of health services and the necessity of a new order of interprofessional relations have been pointed out repeatedly, along with the acknowledgment of the challenges for proactive HHR policies [14, 15].

For health services provided among others in primary, chronic and mental health care, service coordination and interdisciplinary collaboration are an indispensable condition for ensuring adequate service delivery. Since European health care systems share a common concern in what has been labeled as the “epidemic of chronic conditions” ([16]: 143), the need for providing integrated and holistic care and assistance, both within health care and across the interface of health and social services can be expected to grow continuously and rapidly. The demand for a more effective skill mix between health and, increasingly, social professions is hardly controversial. However, the readjustment of the extent of professional autonomy for different professions is, sometimes quite heatedly debated. In the final part of our paper, we will discuss how different models of health care organization relate to service coordination between professions and holistic service delivery.

### Professional jurisdiction as framework for analyzing interprofessional relations

We regard the interface between professions and the regulations governing interprofessional relations a pivotal factor for effective health service delivery. Interprofessional relations can be conceptualized as two related questions. The question of “who does what” in health service delivery is generally discussed as the adequate skill mix between professional groups [14, 17], while the question of “who says you have to” is a matter of professional hierarchy and of ordering interprofessional relations [18, 19].

Both questions are addressed in the concept of professional jurisdiction as developed by Abbott [20], which we use as the theoretical framework for our analysis. In Abbott’s perspective, the jurisdiction for a given set of tasks performed by a profession is not static, but established in the day-to-day work performance ([20]: 33). Professionally performed tasks appear as objective and subjective ones, with the former originating as a professional domain based on technological or organizational qualities. The latter are regarded as culturally allocated to a particular occupational group or profession. Objective tasks may change over time, but much more contested and subject to change are the subjective qualities with professional groups perceived as permanently impinging on the jurisdiction about task performance of other professions ([20]: 39).

The concept of professional jurisdiction has been developed from a historical perspective and provides a valuable framework for conceptualizing the power relations between professional groups. As an example, the development of nursing as a profession is described as the result of a lost power struggle with the medical profession ([20]: 71, 96). However, the concept of professional jurisdiction as presented by Abbott lacks empirically testable parameters that could be used for comparisons between different health systems. For empirical inquiry, specific testable parameters have to be delimited. The analytical value of Abbot’s model when combined with specific analytical parameters has recently been demonstrated in an illustrative study of the nursing profession entering upon the jurisdiction of physicians by obtaining the legal right to prescribe medicines [21, 22]. The study presented a variety of national strategies for regulating the power struggle between health professions in the adjustment of jurisdictional authority. We apply the concept of professional jurisdiction to our analysis of interprofessional relations with a particular concern for integrated and holistic health services, which by their very nature are based on interdisciplinary teamwork and therefore provided by a variety of professions. Regardless of the hierarchical order of professional authority in different health systems, integrated care requires the continuous re-negotiation of professional jurisdictions and the performance of specific subjective tasks of the occupational groups involved. Integrated service delivery to people living with chronic conditions goes far beyond purely medical services [23]. In fact, integrated health services provided for patients with chronic and often multimorbid conditions have to serve a wide continuum ranging from medical services to those supporting activities of daily living. The same is true in community mental health care and rehabilitation. Therefore, the increasing demand for integrated service delivery can be expected to be much more noticeable for nursing and social care services than for medical ones [24–27]. Consequently, a range of subjective tasks organized as medical services under the jurisdiction of physicians can be expected to become challenged increasingly by nursing and social care services. We focus in our analysis therefore particularly on interprofessional relations between physicians, nurses and, to a lesser extent, social care services.

We structured our analysis according to six predefined parameters, which we perceive as major influential forces determining professional jurisdiction and which are regulated differently in different health systems. These parameters were the education and the professional autonomy of nurses, the extent of professional hierarchy, the importance of an adjustment of professional boundaries (skill mix), interdisciplinary teamwork and the cooperation between health and social services. We discuss each of these parameters in detail in the Results section of our paper.

## Methods

### Data

We performed a document analysis of the Health Systems in Transition (HiT) reports published by the European Observatory on Health Systems and Policies on behalf of WHO Europe. The HiT reports aim to describe “the functioning of health systems in countries as well as reform and policy initiatives in progress or under development” [28], and provide at current the arguably most comprehensive, systematic and comparable insight into the organization of European health care systems. Each report is a case study of a European health system, authored by a national expert group. For a number of countries, several revisions of reports have been published. Our data included the most recent HiT report for each country that was published before May 2015. We reviewed a report for each of the 28 EU countries plus Iceland, Norway and Switzerland. For Italy, a separate report describing the Veneto region is available. For the United Kingdom, separate reports are available for the regions of England, Wales, Scotland and Northern Ireland. These separate publications were also included in our analysis. Altogether 35 publications were included in the review.

### Content analysis

We performed a qualitative content analysis of each report. Initially we identified in the table of contents the sections discussing human resource policy, professional jurisdiction and interprofessional relations. Based on a review of these sections, a set of initial codes was developed with word stems that were later applied to lexical searches.

We searched all publications with the word stems of the following terms, which we perceive as indicative for the skill mix and the jurisdiction regulating interprofessional relations.

Jurisdic*; instruct*; gate keep*/gatekeep*; interdisciplin*; multidiscipl*, skill mix/skills mix/mix of skills; human resource*; elder*; morbid*, chronic*.

Each hit on one of these terms was reviewed in its context. Relevant text sequences were coded to the six analytical parameters. Following an analytical strategy described both as meaning categorization ([29]: 190 ff) and as systematic content analysis [30], we assigned nominal- or ordinal-scaled values to the coded sequences. The analytical parameters with their associated values are described below. To enhance the reliability of our analysis, the first and second author assigned the values individually. Where discrepancies appeared, findings were discussed until a consensus was reached. Each HiT report was scored with a single value. When several text sequences in each report were coded to the same analytical parameter, a value was assigned to each passage and the most often assigned value was used for scoring the parameter in question for the country as a whole.

Methodical problems for scoring countries unambiguously were encountered in the case of England were the parameter “education of nurses” according to the HiT report indicated a value of “higher”, meaning that the highest achievable education for nurses ended with a bachelor degree ([31]: 207). Equally, the HiT report for Denmark indicated a value of “intermediate” ([32]: 101). In both cases, this is obviously an inadequate assessment, as PhD programs for nursing exist in all parts of the United Kingdom as well as in Denmark [33, 34]. When available, we therefore supplemented the results of our content analysis with other sources. References to these additional sources are provided in Table 1.

**Table 1:**
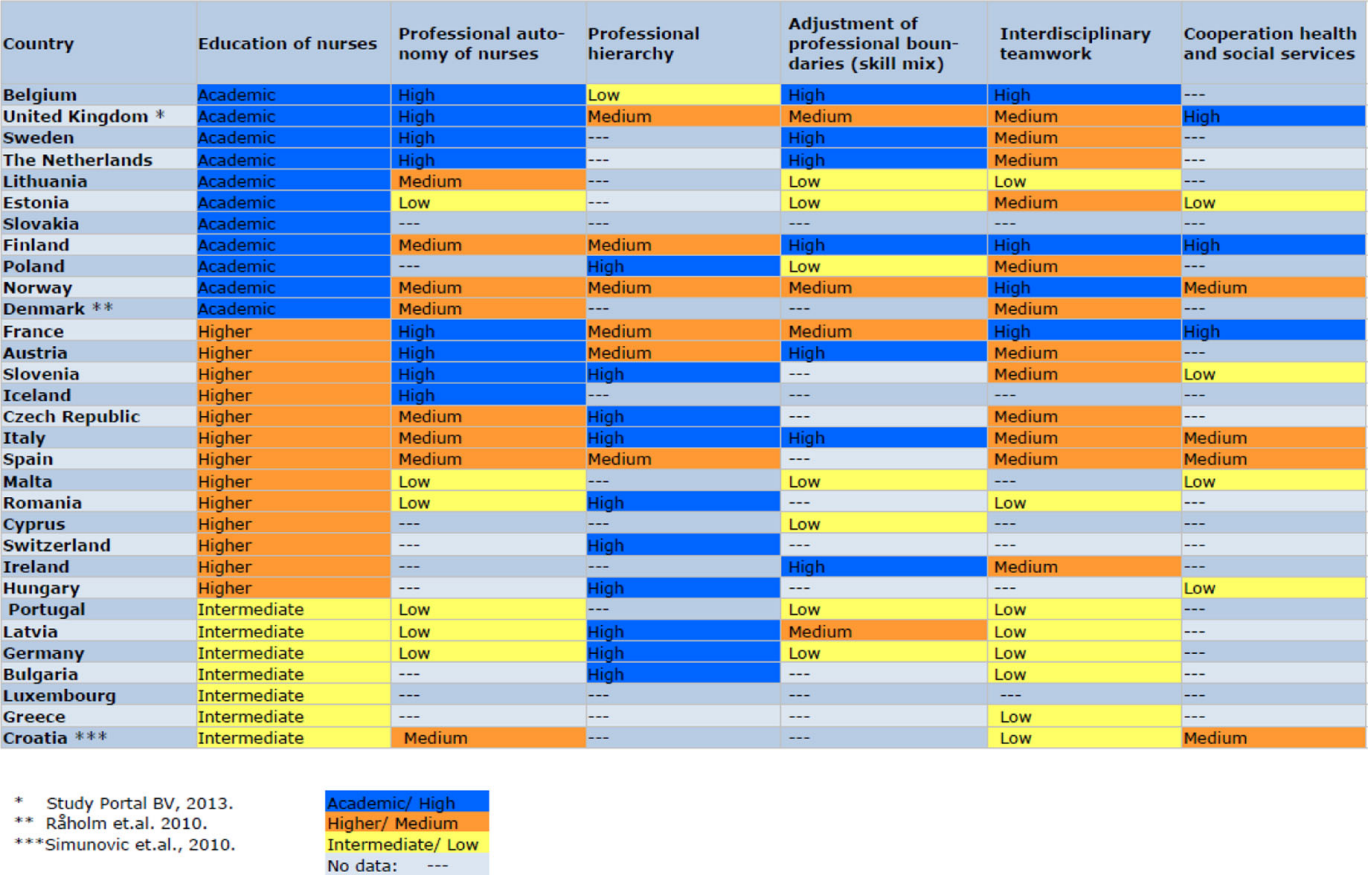
Professional jurisdiction in European health care systems

Lexical search, coding and analysis were performed with MAXQDA; a software supporting qualitative and mixed methods approaches to analysis.^1^

The results of the qualitative content analysis are summarized in Table 1. They provide the point of departure for delimiting three ideal-typical health systems categories, in line with the methodological tradition of Max Weber [35]. Each of these ideal types described in Table 3 has its idiosyncratic features for regulating interprofessional relations and jurisdiction.

## Results

### Parameters with associated values for analysis

#### Education of nurses

For in-depth comparative assessments of health system performance, the distribution of professional roles and jurisdiction in health service delivery are much more informative than the ratio between professional groups. We consider length and level of educational pathways of different professional groups in the health care workforce an important parameter for comparing interprofessional jurisdiction. While education and training for physicians are largely harmonized within the European Economic Association area (EEA), the educational tracks available to other health professions are organized quite heterogeneously. In our analysis, we focused particularly on the education of nurses, because, more than the ratio of physicians and nurses, we perceive the interprofessional relation between these groups as indicative for the capacity of a given health care system for effective care coordination and integrated service delivery. We scored each country according to the highest achievable educational level within nursing. A value of intermediate was assigned to countries where the highest obtainable degree in nursing ended below a bachelor degree; a value of higher to those obtaining a bachelor (BSc or BA), and of academic to those where it is possible to obtain a Masters and/or PhD-degree in nursing.

#### Professional autonomy of nurses

The particular tasks specifically assigned to nurses, and the autonomy they have in the execution of their work varies considerably between European health care systems. We scored country reports with a value of low when the professional autonomy of nurses was presented as very limited, and/or their position was described as subordinated and/or dependent on the authority of other professional groups, and/or where nurses were explicitly prevented from working as self-employed. A value of medium was assigned to reports where the autonomy of nurses within healthcare was described in its own right, but where it was not clearly delimited. The same value was also assigned when the work of nurses was described as partly autonomous from the authority of other professions, and/or where nurses mostly were employed by other health professionals. High was assigned when the professional autonomy of nurses was described in unambiguous terms, and when it was described either as autonomous from the authority of other professions or as protected by a specific legal framework for both employed and self-employed nursing professionals.

#### Professional hierarchy

Physicians represent unequivocally the dominating profession in all European health care systems. As a rule, nurses are the most numerous among the groups of health professions. The professional autonomy of nurses in the performance of their tasks varies as shown. However, the level of professional autonomy is not necessarily indicative for the rigidity of an existing vertical professional hierarchy. We assigned a value of low when a flat hierarchy between occupational groups in the health care sector was indicated or particularly emphasized. Medium was designated to HiT reports when a hierarchical order between occupational groups was indicated and/or the occupational groups were specified, but where it was not indicated who the leading and/or subordinated part in the hierarchy is. High was assigned when a clear hierarchical order between occupational groups was expressed and where the leading profession was mentioned explicitly, and/or where reasons for the hierarchical order were laid out.

#### Adjustment of professional boundaries (skill mix)

The institutional structure in a welfare state, including its health care system and the regulations governing professional jurisdiction is never static. The causes forcing a redistribution of tasks within the health care work force may vary, not the least changes in the demand for the services needed, the availability of particular professional groups and economic constraints require constant adjustments [17, 36]. Nevertheless, in most European health care systems an ongoing discussion takes place about the necessity of a new skill mix. The transfer of responsibilities from physicians to nurses dominates the agenda in several European countries, sometimes, for instance in Germany, vehemently opposed by interest groups of the former [12]. We scored reports with a value of low when the adjustment of professional boundaries was mentioned in passing without specific reasons given for assigning new tasks to another group of health professionals. Also scored as low were reports when a skill mix was described as happening reluctantly, against strong resistance from the profession that performed the task initially, and/or as incomplete in that the originally performing professional group retained a supervising position over the entering professional group. A score of medium was assigned when the reason for the adjustment of professional boundaries was described as a result of an insufficient availability of the necessary work force among the profession that originally performed the task(s), or a financially motivated transfer of tasks to a lower paid professional group. A value of high was assigned when the adjustment of professional boundaries was reasoned for because another profession was more suited for it than the professional group that had performed the task initially.

#### Interdisciplinary teamwork

The need for delivering health services in a coordinated and integrated manner is hardly controversial. However, our analysis revealed that the practical implementation of care-coordination and -integration in several European countries was described as unsatisfactory. While the concept of interdisciplinary teamwork is rather popular in European health policies, it appears with quite different meanings in a variety of national contexts [37]. Instead of the generic use of the term interdisciplinary teamwork, we therefore scored the HiT reports for each country according to the emphasis given to specific examples for how health services delivered by different professions were integrated into a holistic parcel of services to the patient.

Low was assigned when interdisciplinary teamwork was mentioned with no specific professional groups addressed and/or without specific reasons given for different professional groups to work together. Medium was assigned where interdisciplinary teamwork specified the professional groups working together, and/or when reasons for why they are working together, and/or when specific tasks for the team and the team members were mentioned. Medium was also assigned when a vertical professional hierarchy within the team or care delivery and coordination was expressed, and/or when interdisciplinary teamwork was described as pilot projects or temporary trials. High was assigned when, in addition to criteria 1–3 for medium, interdisciplinary teamwork was presented with a consolidated status beyond the status of pilot projects.

#### Cooperation of health and social services

Health and social services have, in most European countries long co-existed alongside each other with limited coordination across the interface of services. The Chronic Care Model, arguably the most influential among recent models for coordinated service delivery to patients living with chronic conditions, addresses for instance exclusively health services, and not the contribution of the variety of social services to the same service recipients [38, 39]. However, the argument for integrating service delivery of both sectors is rapidly gaining momentum [40, 41]. The European Commission has established The European Social Network as a dedicated body for promoting the integration of health and social service delivery.^2^ Still, the cooperation and integration of health and social services is a relatively new issue in the skill mix discussion.

We scored as low when the integration of health and social services was discussed similar to the following text sequence: “… the boundaries between the two sectors are quite unclear. Indeed, service categories can overlap and people can be assigned to the wrong setting, such as when long-term social care for the elderly is provided in acute wards due to the shortage of places in residential homes” ([42]: 155). For this and similar examples, a score of low was assigned when no specific professional groups were addressed, and/or no particular tasks were specified for the services, when the absence of a legal framework enforcing the cooperation between the services was emphasized specifically, and/or the link between health and social services was presented as weak. A value of medium was assigned when specific professional groups working together were pointed out, when reasons were given to why they are working together, when specific tasks that the services had to perform were described, when guidelines (legal or practical) below law status were mentioned or when the link between health and social services was presented as incomplete. A score of high was assigned to reports when, in addition to one of the criteria 1–3 for medium a description of the professional jurisdiction regulating the work of health and social services was mentioned. High was also assigned to cases where the existence of a legal framework specifying conditions for the cooperation between the services was mentioned, and/or when the link between health and social services was presented as strong and consolidated.

### Professional jurisdiction in European health care systems

The results of our analysis are summarized in Table 1. Also provided in this table are references to additional sources rectifying obviously incorrect assumptions based on the analysis of the HiT reports.

We ordered the table according to the distribution of values for the parameter of the education of nurses. The number of countries limiting the education of nurses to an intermediate level is the smallest in our distribution. As most of these countries will streamline the education for different occupational groups, nurses included, according to the guidelines of the European Higher Education Area, their number can be expected to decrease further in the near future.^3^

In the majority of EEA-countries, nursing education is provided in an academic setting, ending with a BA. A considerable number of countries provide possibilities for further academic education, including a research career within nursing.

With the exception of the parameter “Education of nurses”, some HiT reports did not reveal any information to one or several of the analytical parameters. These gaps limit the methodological possibilities for analyzing the data presented in Table 1. We have no intention to draw statistical inferences from the table, nor will we discuss the idiosyncrasies of interprofessional relations and jurisdictions country by country. Instead, we present our interpretations of tendencies that can be observed in Table 2. These tendencies indicate a horizontal consistency of countries scoring respectively high, medium or low on the parameter of the education of nurses on the score received for the other parameters. Therefore, these tendencies provide empirical support for the three ideal-typical models, which we describe in the Discussion of our paper.

**Table 2:**
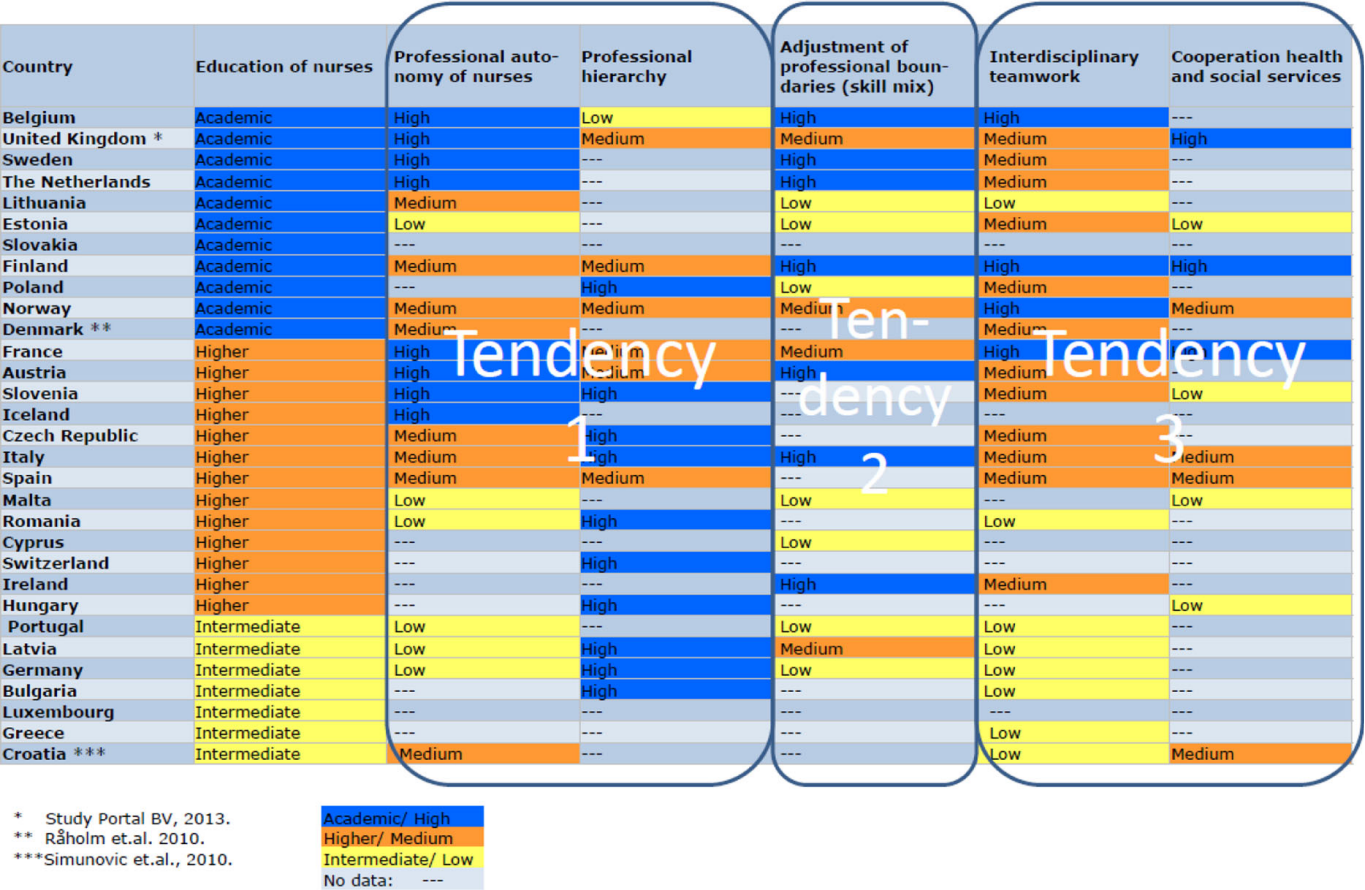
Professional jurisdiction in European health care systems: tendencies

A first tendency regards the position of the nursing profession in the performance of their tasks relative to other professional groups. Marked as tendency 1 in Table 2 it is noticable that academic and higher education for nurses tend to coincide with a reported high and medium degree of professional autonomy for nurses. The opposite can be observed for countries with intermediate and, to a more limited degree, higher educational tracks. Here nursing education tends to coincide with a low degree of professional autonomy. The same is true for the parameter of professional hierarchy. Health systems described with a distinct low and medium level of professional hierarchy are found mainly among those with academic and higher career opportunities for nurses. On the other hand, health care systems with a reported strong professional hierarchy are exclusively among those organizing nursing education in intermediate and higher educational tracks.

Tendency two regards the, in some countries controversial, adjustment of professional boundaries. According to the reviewed HiT reports, this parameter is emphasized most in countries allowing for an academic track in nursing education. On the other hand, a number of eastern European countries, all recently reforming their educational system for nursing, revealed a distinctively low emphasis given to this parameter. No reports with an intermediate nursing education revealed a high emphasis given to the subject, while countries with a higher educational track displayed both high, medium and low emphasis given to an adjustment of professional boundaries.

A third tendency in Table 2 regards the cooperation across professional boundaries, both in the form of interdisciplinary teamwork and across the health and social service interface. The parameter of interdisciplinary teamwork is reported with a strong tendency to high and medium emphasis in countries with academic and higher tracks for nursing education, while it has obviously less relevance in countries with intermediate tracks. Similarly, the relatively new discussion about an integrated approach to care across the interface between health and social services is reported as having a high priority mainly in countries with academic educational tracks for nurses. Low emphasis on the subject on the other hand is spread across countries representing all three educational levels. We must point out that a relatively high number of reports does not discuss the cooperation between health and social services at all. Therefore, the latter tendency should be interpreted with caution.

## Discussion

### An ideal-typical framework for comparing interprofessional relations

In the final part of our paper, we use the results of our analysis to develop a framework of three ideal types for organizing interprofessional relations in health care. We take our point of departure in the methodological tradition of Max Weber who in his classic outline for qualitative methodology described an ideal type as a ‘pure’ type with clearly defined, exaggerated and logically coherent features [35]. The construction of such an ideal type has a dual purpose. On the one hand, it can serve as a tool for measuring empirically observable social phenomena and entities. On the other hand, it allows for statements about causalities delineated from the comparison of ideal type and social phenomena.

We distinguished the ideal types in our model by their particular way of organizing interprofessional relations according to the parameters we have used in our analysis. We have labelled these ideal types respectively as single track, transitional and diversified hierarchical systems.

#### Single track hierarchical systems

This ideal type can be described as a traditional model for regulating interprofessional relations, with the medical profession dominating the jurisdictional order. In this model, while the execution of tasks, either on a day-to-day or on a permanent basis may be delegated from the dominating profession to another, the final authority over both objective and subjective tasks remains firmly in the jurisdiction of the dominant medical profession.

#### Transitional hierarchical systems

The key feature of this ideal type is the promotion of developing effective models for health service delivery, including the development of multiple jurisdictions with negotiable subjective tasks. Transitional hierarchical systems are characterized by a bottom-up culture of multiple emerging models, but lack a regulatory framework ensuring service equity and equality on a national basis.

#### Diversified hierarchical systems

These are characterized by several consolidated jurisdictions, each with a core of objective tasks. At the same time, a large number of subjective tasks are negotiated between members of interdisciplinary teams, partly independent from their affiliation to a particular professional group.

Table 3 summarizes the characteristics of these three ideal types according to the analytical parameters we used in our content analysis.

**Table 3:** Ideal typical categorization of interprofessional relations

	Single track hierarchical systems	Transitional hierarchical systems	Diversified hierarchical systems
Education of nurses	Intermediate.	Higher or academic.	Academic.
Professional jurisdiction	Hierarchical, medically centered professional jurisdiction.	Emerging multiple professional jurisdictions.	Consolidated multiple professional jurisdictions.
Professional hierarchy	Distinct hierarchical professional order.	Indistinct hierarchical order between health professions.	Consolidated co-existing professional hierarchies.
Adjustment of professional boundaries (skill mix)	Reluctantly. Health care work-force outside medicine in sub-ordinated and assisting positions to physicians.	Emerging and expanding fields of independent professional competency for health professions other than physicians.	Consolidated fields of independent professional competency for different professional groups in health and social care.
Interdisciplinary teamwork	Main focus on cooperation between different medical specialties. Subjective tasks of the medical profession are delegated to other professional groups in the healthcare workforce. Interdisciplinary teamwork is managed under the supervision of physiccians.	Unclear professional respon-sibility for subjective tasks. Ad hoc tasks for the professional groups involved. Horizontal professional hierarchy in team leadership, task definition and performance.	Consolidated status in service delivery. Specific objective tasks for the professional groups involved. Team leadership, definition and performance of subjective tasks are a team decision and independent from a vertical professional hierarchy.
Cooperation health and social services	Indistinct. No legal framework enforcing coordination and cooperation.	Pilot projects for specific tasks. Fragmented legal framework for coordination and cooperation.	Formalized with an extensive legal framework for coordination and cooperation.

### Potential impact on service coordination and integrated service delivery

European health care systems have emerged each in its own historical context. As a result, European health care exists in a wide diversity of organizational frameworks for health and social service delivery, chronic care included. As rightly pointed out by Kuhlmann and colleagues [14], HHR-policy and -planning in a European perspective can therefore not aim at developing a one-size-fits-all model. The presented ideal-types represent features that can be found in a variety of national organizational frameworks, and for each ideal-type, certain outcomes can be predicted. The organizational characteristics and expected outcomes of the three ideal-types in the model on service coordination and integrated service delivery as we discuss them in the following can be compared with the organization of interprofessional relations in existing health care systems.

Single-track hierarchical systems have their strength in a well-organized and consolidated professional hierarchy with a clearly delimited catalogue of services that the end-users can expect. Their weakness is a system of care provided primarily with a point of departure in a medical perspective, and with care therefore mainly treated as a question of diagnosis and treatment. Other professional perspectives are either subordinated to the medical profession (such as nursing), or organized separately from the health system (such as social services). The distribution of work between different professional groups remains stagnant, with limited efforts to answer to changes in the demand of service delivery with a new skill mix in the workforce. For the recipients of health services in need of long term and holistic health care, the services not directly related to treatment, and particularly the support to manage the activities of daily living, remain inadequate. Thus, the capability of this ideal type for providing holistic care, and to answer adequately the growing need for coordinated services fields such as primary, chronic or mental health care is limited.

The immediate strength of transitional hierarchical systems is the dynamic development of local models for implementing and testing a variety of strategies for health service delivery. Models are often developed parallel to existing health services with an established distribution of work and an existing legal framework regulating professional jurisdiction. Transitional systems are characterized by an emphasis on interdisciplinary teamwork with continuously adjusted professional boundaries, including teamwork across the interface between health and social services. The dynamic nature of transitional systems is at the same time their strength and weakness. They provide not only an interdisciplinary and dynamic environment for service delivery, but contribute also to a heterogeneous and unpredictable service infrastructure, since the end-users cannot rely on having access to the same service quality geographically and over time. Transitional systems have a high potential for providing effective services, and for meeting the demand for holistic and coordinated services. Their disadvantage is their limited impact on providing these services on a system level. Furthermore, the potential impact is impeded by an incomplete legal framework regulating professional jurisdiction in interdisciplinary work environments.

Within diversified professional hierarchical systems, services are provided within a legal framework of several co-existing professional jurisdictions, including across the interface of health and social services. The skill mix between professions and the transfer of subjective tasks from one professional jurisdiction to another is a continuously debated issue. However, the adjustment of professional boundaries is not necessarily linked to the question of interdisciplinary teamwork. Diversified professional hierarchical systems have a high impact potential for holistic service delivery since several emancipated professional perspectives assert themselves in the tasks benefiting the service recipients. At the same time, the co-existence of multiple jurisdictions entails a danger for causing competition instead of coordination. A possible result of organizing professional jurisdiction with diversified professional hierarchies can therefore be more fragmented instead of interdisciplinary, holistic service delivery.

### Strengths and weaknesses of the study

The strength of our study is the broad comparative base of 31 European health care systems, allowing the empirically supported development of an ideal-typical model of interprofessional relations. The HiT reports provide highly standardized material for comparisons as they are authored according to a common template, including a mandatory section on HHR policies. There are, however, some weaknesses in these data. The time of publication for the most recent HiT report describing the health systems for specific countries varies, with the oldest from 2006 and the most recent from 2015. The organizational framework for each country is therefore reflected as by the time preceding publication. Consequently, more recent and possibly substantial health care reforms are not reflected in the older volumes. Another weakness of the HiT reports regards the discussion of interprofessional relations and jurisdiction. Albeit these are core aspects of HHR policy, the discussion is in some of the reports rather brief and may not have revealed many details to the analytical parameters in our model.

A potential methodological weakness regards the search terms we used in our content analysis. These may in some instances not have been sensitive enough to capture all aspects of interprofessional relations discussed in the HiT reports. A final weakness regards the number of HiT reports that did not reveal any data at all to one or several of the analytical parameters. This does not diminish the value of the final ideal-typical model for comparisons of interprofessional relations, but it should be kept in mind that the information provided to different aspects of interprofessional relations varies between the HiT reports to specific national health systems.

## Conclusions

Current European health policies emphasize heavily the concepts of holistic service delivery, integrated care, and interdisciplinary teamwork. The successful implementation of these concepts depends on a more effective skill-mix among health professions and proactive HHR policies.

This study demonstrated the problematic nature of precisely measurable performance indicators for the comparison of interprofessional relations in different health care systems. Based on the comparison of 31 European health care systems we introduced an ideal-typical model as a means for in-depth comparisons of interprofessional relations and the skill mix in health care. We demonstrated the use of the model by assessing the potential impact of each ideal type on service coordination and integrated service delivery. The presented model provides the means for comparing the features of the three ideal types with the skill mix and interprofessional relations in specific health care systems. Thus, the model opens for in-depth comparative assessments of health care systems beyond of what is possible with directly comparable, but generic performance indicators.

## Abbreviations

EEA: European Economic Association area
HHR: Health human resource
HiT: Health Systems in Transition
WHO: World Health Organization

## Footnotes

1
http://www.maxqda.com/

2
http://www.esn-eu.org/news/230/index.html.

3
http://ec.europa.eu/education/policy/higher-education/bologna-process_en.htm; accessed August 12, 2015.
